# Prolyl isomerase Pin1 plays an essential role in SARS-CoV-2 proliferation, indicating its possibility as a novel therapeutic target

**DOI:** 10.1038/s41598-021-97972-3

**Published:** 2021-09-17

**Authors:** Takeshi Yamamotoya, Yusuke Nakatsu, Machi Kanna, Shun Hasei, Yukino Ohata, Jeffrey Encinas, Hisanaka Ito, Takayoshi Okabe, Tomoichiro Asano, Takemasa Sakaguchi

**Affiliations:** 1 Department of Medical Chemistry, Graduate School of Biomedical and Health Sciences, Hiroshima University, 1-2-3 Kasumi, Minami-ku, Hiroshima, 734-8551 Japan; 2 Anenti Therapeutics Japan, Inc., 4-3 Yamaashiya-cho, Ashiya, 659-0082 Japan; 3 School of Life Sciences, Tokyo University of Pharmacy and Life Sciences, 1432-1 Horinouchi, Hachioji, Tokyo 192-0392 Japan; 4 Drug Discovery Initiative, The University of Tokyo, 7-3-1 Hongo, Bunkyo-ku, Tokyo, 113-0033 Japan; 5 Department of Virology, Graduate School of Biomedical and Health Sciences, Hiroshima University, 1-2-3 Kasumi, Minami-ku, Hiroshima, 734-8551 Japan

**Keywords:** Infectious diseases, Target identification, SARS-CoV-2, Drug development

## Abstract

Novel coronavirus disease 2019 (COVID-19) has emerged as a global pandemic with far-reaching societal impact. Here we demonstrate that Pin1 is a key cellular molecule necessary for severe acute respiratory syndrome coronavirus 2 (SARS-CoV-2) propagation. In this study, siRNA-mediated silencing of Pin1 expression markedly suppressed the proliferation of SARS-CoV-2 in VeroE6/TMPRSS2 cells. In addition, several recently generated Pin1 inhibitors showed strong inhibitory effects on SARS-CoV-2 proliferation, measured by both viral mRNA and protein synthesis, and alleviated the cytopathic effect (CPE) on VeroE6/TMPRSS2 cells. One compound, termed H-77, was found to block SARS-CoV-2 proliferation at an EC_50_ below 5 μM regardless of whether it was added to the culture medium prior to or after SARS-CoV-2 infection. The inhibition of viral N protein mRNA synthesis by H-77 implies that the molecular mechanism underlying SARS-CoV-2 inhibition is likely to be associated with viral gene transcription or earlier steps. Another Pin1 inhibitor, all-trans retinoic acid (ATRA)—a commercially available drug used to treat acute promyelocytic leukemia (APL) and which both activates the retinoic acid receptor and inhibits the activity of Pin1—similarly reduced the proliferation of SARS-CoV-2. Taken together, the results indicate that Pin1 inhibitors could serve as potential therapeutic agents for COVID-19.

## Introduction

In December 2019, an outbreak of pneumonia occurred in Wuhan, China, caused by a virus later designated as severe acute respiratory syndrome coronavirus 2 (SARS-CoV-2)^1^. Following the outbreak, a global pandemic of SARS-CoV-2 infections has seriously disturbed daily life and economic activities, and intense efforts worldwide have been initiated to find effective therapies and vaccines to combat the pandemic. Notably, the lethality mortality rate of coronavirus disease 2019 (COVID-19) is higher in subjects with obesity, diabetes mellitus^2^, and advanced age, in contrast to the high mortality rate in the young population observed in the case of the 1918 Spanish influenza pandemic^3^. The prevailing hypothesis for this high lethality in a subset of individuals is that it is attributable to the more serious effects of a cytokine storm induced by SARS-CoV-2 in patients with chronic inflammatory status related to underlying obesity or diabetes mellitus^4,5^.

Over the past several years, our research group has focused on elucidating the role of peptidyl-prolyl isomerase Pin1 in metabolic regulation^6^. There are three groups of peptidyl-prolyl isomerases (PPIases): the FKBP, Cyclophilin, and Parvulin families (Pin1 and Par14)^7^. Pin1 is unique among the PPIases in that it binds to pSer/pThr-Pro motifs and functions by modulating the enzymatic activity, protein stability, or subcellular localization of target proteins by catalyzing a cis-to-trans orientation of proline in its substrate’s protein structure. Many studies have revealed roles of Pin1 in cancers, metabolism, and Alzheimer’s disease^8,9^. Evidence suggests that Pin1 expression in cancer cells is closely related to the degree of their malignancy, as Pin1 enhances cell proliferation and inhibits apoptosis^10,11^. Nevertheless, Pin1 is not indispensable for the survival or growth of normal cells. Pin1 KO mice are born and become mature without any defects in size and appearance^12,13^.

We have observed that Pin1 expression levels are markedly increased in several tissues including the liver, muscle, adipose tissue, and kidney in obese or diabetic mice^6^. Interestingly, Pin1 reportedly accelerates the proliferation of several viruses, although the molecular mechanism underlying Pin1-induced promotion of virus proliferation seems to differ among virus types^14–18^. Taken together, we speculate that the increased Pin1 expression in obese or diabetic patients may be involved in the rapid progress and/or severity of infection with SARS-CoV-2.

## Results

### Essential role of Pin1 in SARS-CoV-2 proliferation

**Figure 1:**
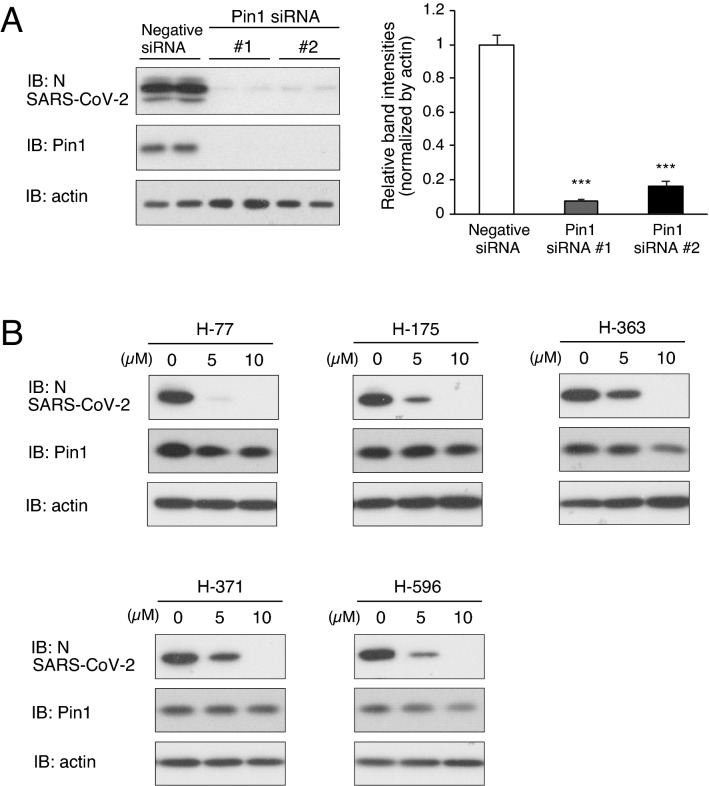
Both Pin1 knockdown and treatment with Pin1 inhibitors significantly reduced SARS-CoV-2 proliferation in VeroE6/TMPRSS2 cells. (**A**) VeroE6/TMPRSS2 cells were transfected with either Pin1 siRNA or negative siRNA, and after three days the cells were infected with SARS-CoV-2 at an input MOI of 0.01 followed by incubation for 24 h (n = 3–4). Cell lysates were processed for Western blotting with SARS-CoV-2 N, Pin1 and actin antibodies, and the bands are shown (Duplicate in the figure). The density of the N protein bands was quantitated, and relative density is shown in the graph with Negative siRNA as 1.0. ***P < 0.001, Mann–Whitney U test, compared with Negative siRNA. (**B**) The proliferation of SARS-CoV-2 in VeroE6/TMPRSS2 cells pretreated with 5 or 10 µM of each Pin1 inhibitor or 0 µM (DMSO). The cells were pretreated with a drug 2 h before infection and infected with SARS-CoV-2 at an MOI of 10, followed by incubation for 8 h in the presence of the drug. Cell lysates were processed for Western blotting using SARS-CoV-2 N, Pin1, and actin antibodies. Full-length blots are presented in Supplementary Fig. 3.

We first examined the contribution of Pin1 to SARS-CoV-2 proliferation using VeroE6/TMPRSS2 cells, which are highly susceptible to SARS-CoV-2 infection due to their constitutive expression of transmembrane serine protease TMPRSS2^19^. Initially, we examined the effect of siRNA-mediated suppression of Pin1 expression on SARS-CoV-2 proliferation in VeroE6/TMPRSS2 cells. Treatment of the cells with either of two Pin1 siRNAs markedly reduced the expression of Pin1 protein and reduced the proliferation of SARS-CoV-2 in the cells as assessed by SARS-CoV-2 nucleocapsid (N) protein levels detected in the cell lysates (Fig. 1A). Notably, the degree of reduction was more pronounced in our study than in a previous study in which feline coronavirus replication was partially suppressed by treatment with Pin1 siRNA^14^. Subsequently, we investigated the effect of Pin1 inhibitors on SARS-CoV-2 proliferation.

### Inhibition of SARS-CoV-2 proliferation by Pin1 inhibitors

**Figure 2:**
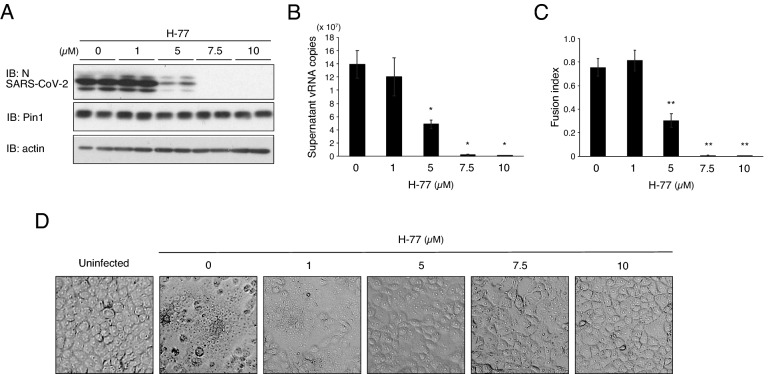
Pin1 inhibitor H-77 inhibits SARS-CoV-2 proliferation in a concentration-dependent manner. VeroE6/TMPRSS2 cells were pretreated with the indicated concentrations of H-77 for 2 h, and were subjected to SARS-CoV-2 infection (MOI of 0.01) followed by incubation for 24 h in the presence of the same concentrations of H-77. (**A**) Western blotting of cell lysates (Duplicate in the figure) with SARS-CoV-2 N, Pin1, and actin antibodies. Full-length blots are presented in Supplementary Fig. 4. (**B**) vRNA copies contained in 5 µL of the supernatant were quantified by RT-qPCR using authorized SARS-CoV-2 N primers (n = 3). (**C**) Fusion index calculated by counting numbers of cells and nuclei of infected cells after Giemsa straining. Approximately 100 nuclei and cell number per field were counted for each of five fields. Error bars indicate standard deviation. *P < 0.05, **P < 0.01, Mann–Whitney U test, compared with 0 µM (DMSO). (**D**) Representative images of SARS-CoV-2-infected VeroE6/TMPRSS2 cells at 24 h post-infection with the indicated concentrations of H-77.

We have recently developed many novel compounds with Pin1 inhibitory activity, and they were experimentally characterized for their effects on SARS-CoV-2. Our experiments revealed that at least 20 of these compounds exhibit a strong suppressive effect on SARS-CoV-2 proliferation at a concentration of 10 μM. The chemical structures and the results for five representative compounds are shown in Table 1 and Fig. 1B, respectively. Studies on the viability of infected cells revealed that a cytopathic effect (CPE), syncytium formation, of VeroE6/TMPRSS2 cells by SARS-CoV-2 was also almost completely prevented by the addition of Pin1 inhibitors to the culture medium (The results of H-77 are shown in Fig. 2C,D.).

**Table 1:**
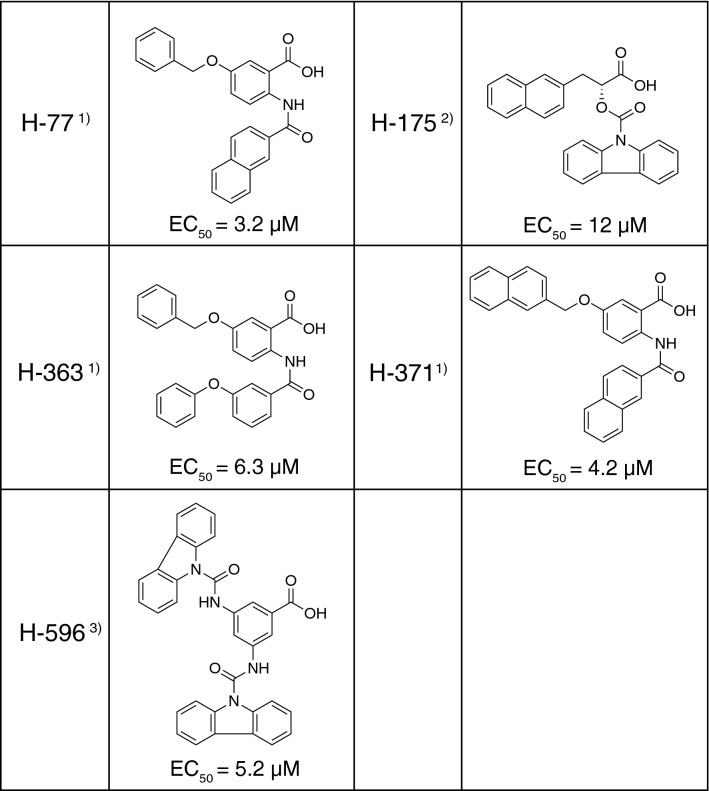
Pin1 inihibitors that inhibited SARS-CoV-2 proliferation.

1) T. Asano, Y. Nakatsu, H. Ito, T. Okabe, WO/2019/031,472.

2) T. Asano, Y. Nakatsu, H. Ito, T. Okabe, WO/2018/101,329.

3) T. Asano, Y. Nakatsu, H. Ito, T. Okabe, JP2020-191,046.

The 50% effective concentration (EC_50_) was calculated using the virus production at different drug concentrations as an indicator (Supplementary Fig. 1), and the values were entered in Table 1. Since H-77 showed the smallest value of 3.2 µM, more detailed studies were performed using H-77 as a potent suppressor of SARS-CoV-2 proliferation.

The concentration-dependent effect of H-77 against SARS-CoV-2 was shown by measuring viral protein levels in VeroE6/TMPRSS2 cells (Fig. 2A) or viral RNA isolated from the culture medium (Fig. 2B). Membrane fusion, a CPE caused by viral infection, became less apparent as the drug concentration was increased to 5 μM and was almost absent at concentrations above 7.5 μM (Fig. 2D). This trend was evident in the fusion index, which quantifies the degree of membrane fusion (Fig. 2C). Considering the data obtained by disrupting Pin1 activity with siRNA and various Pin1 inhibitory compounds, it can be concluded that Pin1 is essential for SARS-CoV-2 proliferation.

**Figure 3:**
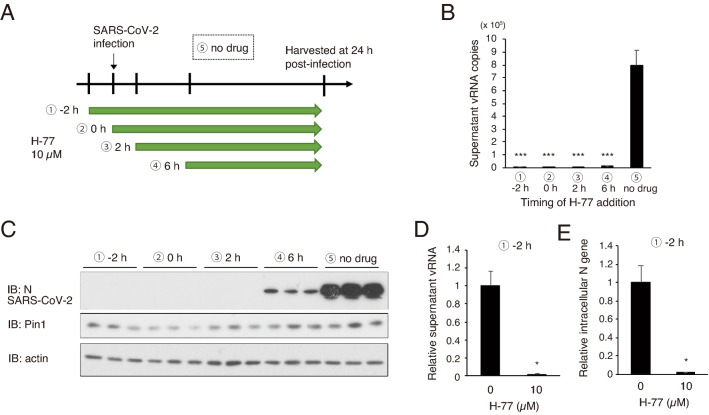
Effect of H-77 treatment timing on growth inhibition of SARS-CoV-2. VeroE6/TMPRSS2 cells were infected with SARS-CoV-2 (MOI of 0.01), and H-77 (final concentration 10 µM) was added at different times as indicated. Cells and supernatants were harvested 24 h after infection. (**A**) Schematic diagram showing the timing of drug addition and experimental conditions. ① 2 h before viral infection, ② simultaneously with a viral infection, ③ 2 h after infection, ④ 6 h after infection, and ⑤ without drug addition. (**B**) Copy number per PCR reaction (8 µl) of vRNA in the culture supernatant 24 h after infection (n = 3). Error bars indicate standard deviation. ***P < 0.001, Mann–Whitney U test, compared with ⑤ no drug. (**C**) Western blotting of cell lysates with SARS-CoV-2 N, Pin1, and actin antibodies (Triplicate in the figure). Full-length blots are shown in Supplementary Fig. 4. (**D**, **E**) VeroE6/TMPRSS2 cells were pretreated with 10 µM H-77 under the condition described in ① (starting from 2 h before infection), and RNA was extracted from the cells 24 h after viral infection. RT-qPCR was used to measure vRNA in the supernatant collected at the same time (n = 3) (**D**) and intracellular N genes (mainly mRNA, adjusted for total RNA content) (n = 3) (**E**). Error bars represent standard deviation. *P < 0.05, Mann–Whitney U test.

We therefore next investigated whether H-77 can exert its inhibitory effect even when added at the same time as the SARS-CoV-2 infection or after in order to determine its applicability as a therapeutic agent (Fig. 3A). Our results showed that H-77 almost completely blocked SARS-CoV-2 proliferation when added 2 h after infection and showed a weaker but still significant inhibitory effect when added 6 h after infection (Fig. 3B,C). The amount of genomic RNA released from the cells was significantly reduced by H-77 treatment (Fig. 3D). In addition, intracellular viral N mRNA was significantly reduced, although some genome RNA was also mixed in (Fig. 3E), providing evidence that H-77 inhibits viral proliferation at the viral RNA transcription step or earlier.

**Figure 4:**
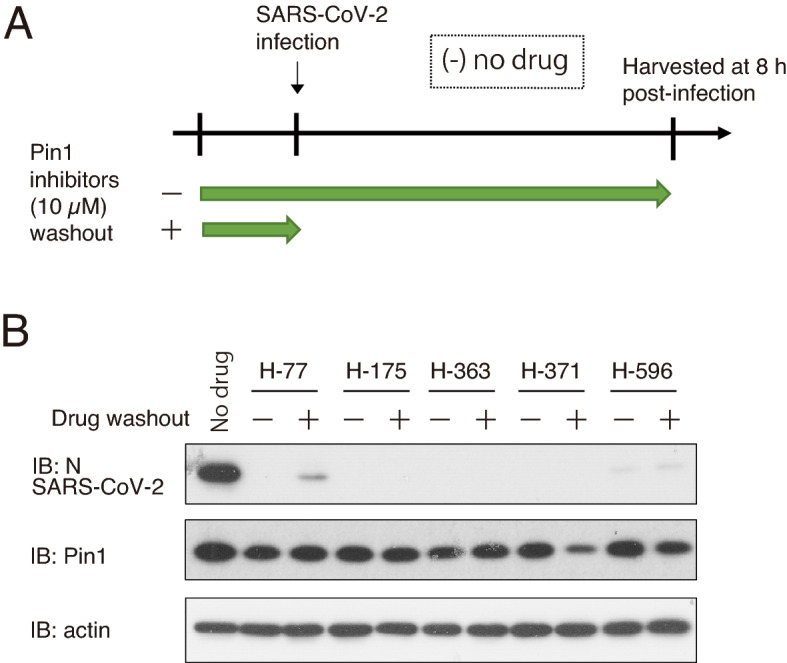
Inhibition of SARS-CoV-2 proliferation by Pin1 inhibitors was not abolished upon their removal before SARS-CoV-2 infection. VeroE6/TMPRSS2 cells were pretreated with 10 μM Pin1 inhibitors for 2 h. The Pin1 inhibitors in the medium were either washed out (+) or retained ( −), and then the cells were infected with SARS-CoV-2 at an MOI of 10 followed by incubation for 8 h. (**A**) Schematic diagram showing the incubation period with Pin1 inhibitors. (**B**) Western blotting of cell lysates with SARS-CoV-2N, Pin1, and actin antibodies. Full-length blots are presented in Supplementary Fig. 4.

Five potent Pin1 inhibitors, including H-77, were applied for 2 h before virus infection, followed by washing out the Pin1 inhibitors before virus infection. N-protein synthesis of SARS-CoV-2 was strongly inhibited even after washout (Fig. 4A,B). These results indicate that the Pin inhibitor is effective if the cells are pretreated immediately before virus infection.

### Inhibition of viral replication by a medical agent

**Figure 5:**
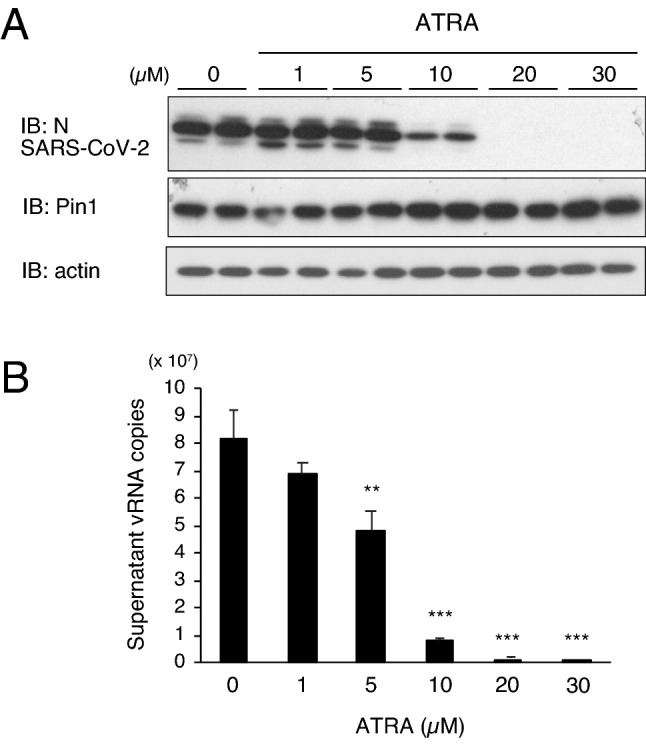
ATRA with Pin1 inhibitory activity also inhibits SARS-CoV-2 proliferation. VeroE6/TMPRSS2 cells were pretreated with indicated concentrations of ATRA for 2 h and subsequently infected with SARS-CoV-2 (MOI of 0.01) followed by incubation for 24 h. (**A**) Western blotting of cell lysates with SARS-CoV-2N, Pin1, and actin antibodies (Duplicate in the figure). Full-length blots are presented in Supplementary Fig. 4. (**B**) Copy number per PCR reaction (8 µl) of vRNA in the culture supernatant 24 h after infection (n = 3). Error bars represent standard deviation. **P < 0.01, ***P < 0.001, Mann–Whitney U test, compared with 0 µM (DMSO).

At present, no highly specific Pin1 inhibitor is commercially available for either medical or experimental purposes. Although Juglone is the most commonly used Pin1 inhibitor compound for basic research, it reportedly binds to and inhibits the activity of many proteins, including tubulin, in addition to Pin1, and it was found that VeroE6/TMPRSS2 cells were unable to survive incubation with 2 μM Juglone for more than 12 h. As an alternative, we tested all-trans retinoic acid (ATRA), an agonist of the retinoic acid receptor (RAR) that is used medically to treat acute promyelocytic leukemia (APL) and was recently reported to inactivate Pin1 isomerase activity^20^. The activities of ATRA as an RAR agonist and a Pin1 inhibitor both contribute to the suppression of APL cell growth^20^. As a result, it was found that ATRA similarly suppressed SARS-CoV-2 proliferation as shown by marked reductions in protein and viral RNA levels in a concentration-dependent manner (Fig. 5A,B) and alleviated its CPE (Supplementary Fig. 2), although the EC_50_ of ATRA (17.9 µM, Supplementary Fig. 1) was higher than that of H-77 (3.2 µM).

## Discussion

This study is the first study to demonstrate the essential role of Pin1 in SARS-CoV-2 proliferation and the possibility of Pin1 inhibition as a promising therapy against COVID-19. In the present study, the knockdown of Pin1 by siRNA inhibited the growth of SARS-CoV-2. On the other hand, we performed overexpression experiments using a Pin1 expression plasmid, but the results were unclear. Pin1 is upregulated in VeroE6/TMPRSS2 cells, which may have made it difficult to get results from the overexpression experiment. Pin1 tends to be upregulated in cancer cells and cells with active proliferation^10,11^. The inhibitory activities of our Pin1 inhibitors, H-77, H-175, H-363, H-371 and H-596, have been confirmed to inhibit Pin1 enzyme activity in an in vitro PPIase assay using recombinant Pin1 protein (Supplementary Table 1). However, potential non-specific effects on other proteins such as other PPIase enzymes or kinases have not yet been sufficiently ruled out. On the other hand, ATRA reportedly inhibits the activity Pin1 but does not affect the activity of FKBP or cyclophilin^20^. Thus, our results using Pin1 siRNAs and ATRA strongly support the involvement of Pin1 rather than that of other PPIases in the proliferation of SARS-CoV-2. We hypothesize that it is highly likely that the inhibitory effects of our five compounds on SARS-CoV-2 proliferation are mediated specifically through Pin1 inhibition, although the possibility of the existence of an additional mechanism(s) cannot be ruled out.

Interestingly, Pin1 has also been reported to enhance the proliferation of several other viruses including human immunodeficiency virus type 1 (HIV-1)^15^, hepatitis C virus (HCV)^16^, Epstein-Barr virus (EBV)^17^, human T-lymphotropic virus type 1 (HTLV-1)^18^, and feline coronavirus^14^. The molecular mechanisms underlying Pin1-induced enhancement of viral proliferation can be largely divided into two mechanisms. One is mediation by enhanced production of oncogenic or inflammatory proteins in the host cells via association of Pin1 with cyclin D1, NF-kB, and Tax^18^. The other is direct involvement of Pin1 in various aspects of the life cycle of viruses such as core exuviation, genome integration, and RNA or DNA replication. For example, Pin1 has been shown to contribute to the uncoating of the HIV-1 core, reverse transcription of the RNA genome, and integration of HIV-1 genomic DNA into chromosomes^15^. In the case of EBV, Pin1 binds to the subunit of DNA polymerase, termed BALF5, and enhances replication^17^. Our results suggest that Pin1 plays a critical role in viral gene transcription or earlier steps after invasion of SARS-CoV-2 into cells and thus appears to be indispensable for SARS-CoV-2 proliferation. Further studies are necessary to identify the target protein of Pin1 and its functions in the life cycle of SARS-CoV-2.

In conclusion, our study clearly showed an essential role of Pin1 in SARS-CoV-2 proliferation. Accordingly, the use of Pin1 inhibitors might be an effective therapy against COVID-19. Our study also indicated the necessity for optimizing and/or developing novel compounds with both potent Pin1 inhibitory activity and high specificity.

## Methods

### Cell culture

VeroE6/TMPRSS2 cells (African green monkey kidney-derived cells expressing human TMPRSS2, purchased from the Japanese Collection of Research Bioresources (JCRB) Cell Bank, JCRB1819) were maintained in Dulbecco’s Modified Eagle’s Medium (DMEM) containing 10% fetal bovine serum (FBS) and 1 mg/mL G418 at 37 °C in 5% CO_2_. For siRNA treatment, VeroE6/TMPRSS2 cells were transfected with either negative siRNA (QIAGEN) or Pin1 siRNA (Invitrogen) using RNAiMAX (Invitrogen) according to the manufacturer’s protocol and subjected to SARS-CoV-2 infection 3 days later. Pin1 siRNA1: CCG UGU UCA CGG AUU CCG GCA UCC A. Pin1 siRNA2: GCC CUG GAG CUG AUC AAC GGC UAC A.

### Pin1 inhibitors

Chemical structures of Pin1 inhibitors termed H-77, H-175, H-363, H-371 and H-593 are shown in Table 1. These Pin1 inhibitors inhibit isomerase activity by more than 80% at a concentration of 20 μM, based on an in vitro* assay* using recombinant Pin1 protein. However, it should be noted that the results of such an in vitro assay usually differ significantly from the results obtained by in vivo experiments. The compounds were solubilized in DMSO. Before the infection experiments, the culture medium of VeroE6/TMPRSS2 cells was changed to DMEM without FBS and G-418, and virus and/or Pin1 inhibitors were added at the indicated titer or concentrations.

### SARS-CoV-2 infection

The SARS-CoV-2/JP/Hiroshima-46059T/2020 strain (accession number MZ853926), which was isolated from a cluster infection in Hiroshima^21^, was used. To prepare virus suspensions*,* VeroE6/TMPRSS2 cells were infected with the virus and incubated in DMEM. The virus titer was determined by the standard 50% tissue culture infectious dose (TCID_50_) method and expressed as TCID_50_/ml as described previously^22^. SARS-CoV-2 infection was performed in the BSL3 facility of Hiroshima University. Unless otherwise noted, VeroE6/TMPRSS2 cells were inoculated with SARS-CoV-2 at an input multiplicity of infection (MOI) of 0.01 followed by incubation for 24 h or an MOI of 10 followed by incubation for 8 h.

### Membrane fusion and fusion index

Vero cells infected with SARS-CoV-2 at an MOI of 0.01 with or without a Pin1 inhibitor were observed with an inverted microscope (ZEISS Axiovert 40/CFL) and photographed with a microscope camera (INOCAM-HD2; Inohara, Hiroshima, Japan) on the following day. The cells were then washed with PBS, fixed with methanol, and stained with Giemsa staining solution. The fusion index was calculated as [1-(number of cells/number of nuclei)] as described previously^23^. Approximately 100 nuclei and cell number per field were counted, and the average fusion index of five fields was calculated.

### Western blotting

Drug-treated infected cells (ca. 1.0 × 10^5^ cells) in 24-well plates were lysed in 70 µl of 1 × SDS sample buffer [50 mM Tris–HCl, pH 6.8, 2% sodium dodecyl sulfate (SDS), 10% glycerol, 840 mM 2-mercaptoethanol, bromophenol blue]. After heat denaturation at 95 °C for 3 min, 10 µl of the sample was was fractionated by SDS–polyacrylamide gel electrophoresis (PAGE). Proteins were transferred to PVDF membranes, and subjected to immunoblotting using Supersignal West Pico PLUS Chemiluminescent Substrate (Thermo Scientific, Waltham, MA, USA). The antibodies used were from GeneTex (Irvine, CA, USA) [SARS-CoV/SARS-CoV-2 (COVID-19) nucleocapsid antibody (GTX632269)] and Santa Cruz Biotechnology (Dallas, TX, USA) [βand Pin1 (sc-46660)]. Chemiluminescence was detected on x-ray film (Super RX; FUJIFILM Medical Co., Lid., Tokyo, Japan), and the film was read by a scanner (CanoScan LiDE 220; Canon, Tokyo, Japan) after development. Quantification of the band density was performed by the Image J software.

### Quantitative real-time PCR

For quantification of SARS-CoV-2 in the supernatant of infected cells, RNA extraction and RT-qPCR were performed using the SARS-CoV-2 Direct Detection RT-qPCR kit according to the manufacturer's instructions (Takara Bio Inc., Kusatsu, Japan); Eight µl of the supernatant was used in a 20 µl-reaction. The RNA genome quantity was determined using the new coronavirus positive control RNA (Nihon Gene Research Laboratory, Sendai, Japan) as a standard. Cellular RNA in the infected VeroE6/TMPRSS2 cells was prepared using the Maxwell RSC instrument (Promega Corp., Madison, WI) according to the manufacturer’s protocol. RT-qPCR for specific amplification of the N gene of SARS-CoV-2 was performed using One Step PrimeScript III RT-qPCR mix (Takara Bio Inc.) according to the manufacturer's protocol. The Primer/Probe Set (2019-n) (Takara Bio Inc.) contains two primer sets, N and N2, both annealing to the N gene of SARS-CoV-2. Thermal cycling was carried out as follows: reverse transcription at 52 °C for 5 min, initial denaturation at 95 °C for 10 s, 45 cycles of denaturation at 95 °C for 5 s, and a final annealing/extension at 60 °C for 30 s. LightCycler 480 System II (Roche Diagnostics K. K., Basel, Switzerland) was used as the instrument for the PCR reaction.

## Supplementary Information

Supplementary Information 1.

Supplementary Information 2.

Supplementary Information 3.

Supplementary Information 4.

Supplementary Information 5.
