# Supplementary figures and images for: Prolyl isomerase Pin1 plays an essential role in SARS-CoV-2 proliferation, indicating its possibility as a novel therapeutic target
Source: Sci Rep. 2021 Sep 17;11:18581. doi: 10.1038/s41598-021-97972-3 (PMC8448864; doi:10.1038/s41598-021-97972-3)

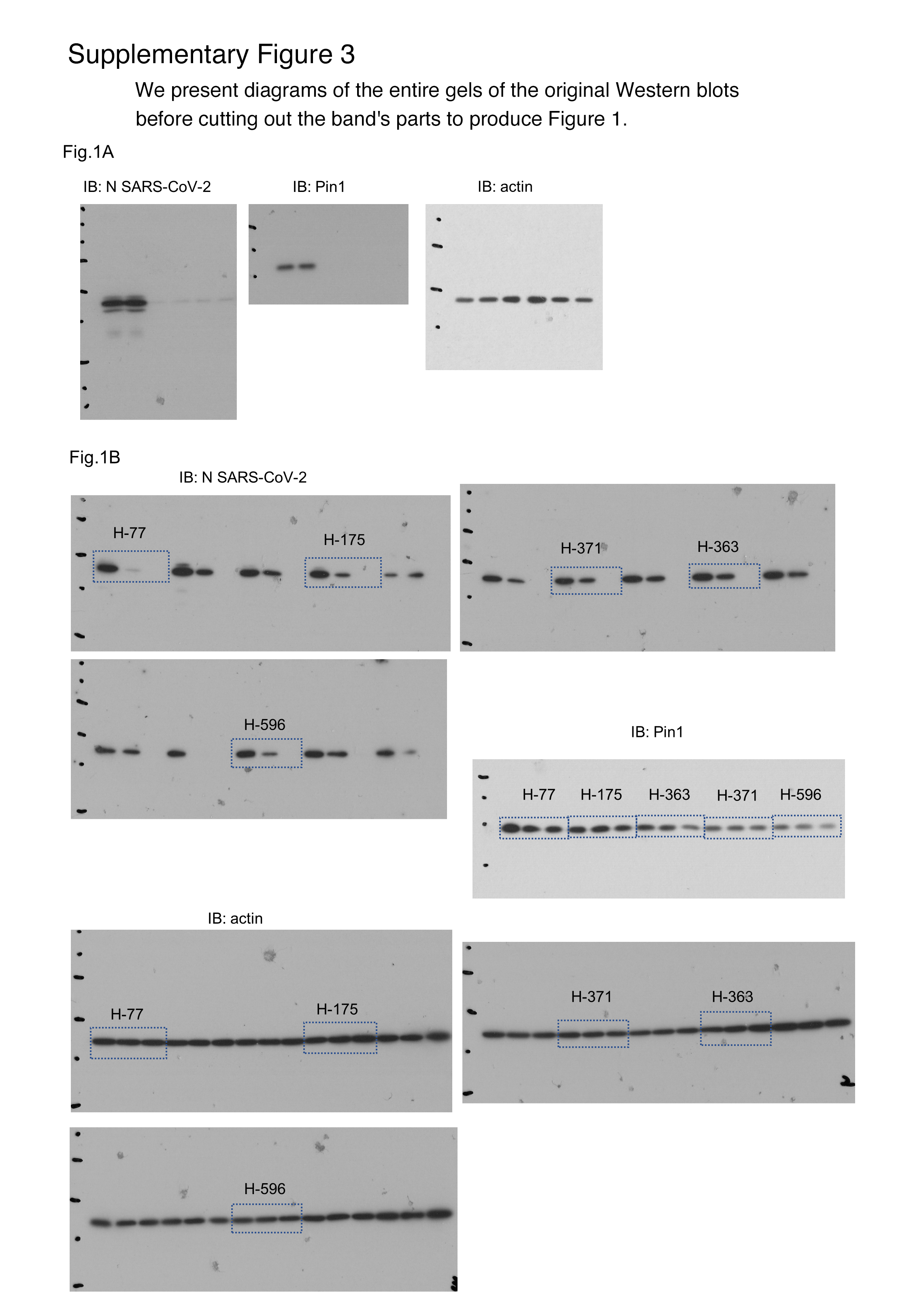

Supplement: Supplementary file 3 — Supplementary Information 3. [file 41598_2021_97972_MOESM3_ESM.tif]

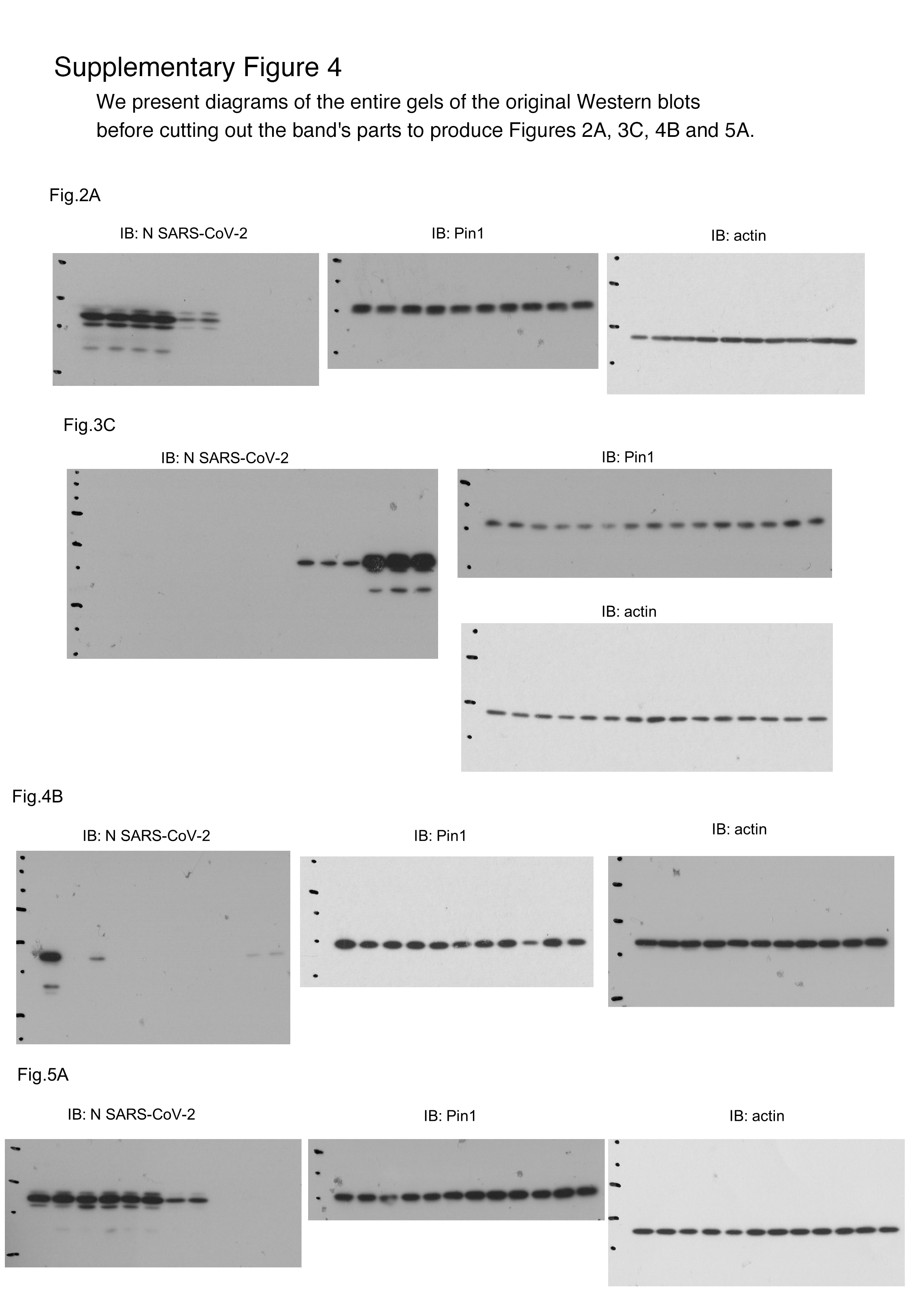

Supplement: Supplementary file 4 — Supplementary Information 4. [file 41598_2021_97972_MOESM4_ESM.tif]
