# Supplementary material for: Prolyl isomerase Pin1 plays an essential role in SARS-CoV-2 proliferation, indicating its possibility as a novel therapeutic target
Source: Sci Rep. 2021 Sep 17;11:18581. doi: 10.1038/s41598-021-97972-3 (PMC8448864; doi:10.1038/s41598-021-97972-3)
Supplement: Supplementary file 5 — Supplementary Information 5. [file 41598_2021_97972_MOESM5_ESM.docx]

Supplementary Table 1. *In vitro* Pin1 inhibitory activity of five drugs.

————————————————————————————

ID concentration (µM) Inhibition (%)

————————————————————————————

H-77 20 82

2 67

H-175 20 89

H-371 2 80

H-363 20 82

H-596 1 13

10 101

————————————————————————————

The *in vitro* Pin1 inhibitory activity of the drugs was determined by the method of Janowski et al. (Janowski, B. et al. Anal. Biochem. 252, 199-307, 1997), and the percent inhibition at a specific concentration is shown in the table. The measurements were performed by Eurofins Selcia Ltd. (Ongar, UK).
